# Taurocholic acid is an active promoting factor, not just a biomarker of progression of liver cirrhosis: evidence from a human metabolomic study and in vitro experiments

**DOI:** 10.1186/s12876-018-0842-7

**Published:** 2018-07-11

**Authors:** Zhimin Liu, Zhifeng Zhang, Mei Huang, Xiaoping Sun, Bojia Liu, Qiyang Guo, Qingshan Chang, Zhijun Duan

**Affiliations:** 1 Second department of Gastroenterology, First Affiliated Hospital of Dalian Medical University, Dalian, 116011 China; 2 The Sixth People’s Hospital of Dalian, Dalian, 116021 China

**Keywords:** Taurocholic acid, Liver cirrhosis, Hepatic stellate cell, Metabolomics

## Abstract

**Background:**

Previous studies have indicated that bile acid is associated with progression of liver cirrhosis. However, the particular role of specific bile acid in the development of liver cirrhosis is not definite. The present study aims to identify the specific bile acid and explore its possible mechanisms in promoting liver cirrhosis.

**Methods:**

Thirty two cirrhotic patients and 27 healthy volunteers were enrolled. Age, gender, Child-Pugh classification and serum of patients and volunteers were collected. Liquid chromatography tandem mass spectrometry (LC-MS) was utilized to determine concentrations of 12 bile acids in serum. Principal component analysis, fold change analysis and heatmap analysis were used to identify the most changed bile acid. And pathway analysis was used to identify the most affected pathway in bile acid metabolism. Spearman rank correlation analysis was employed to assess correlation between concentrations of bile acids and Child-Pugh classification. Hepatic stellate cells (LX-2) were cultured in DMEM. LX-2 cells were also co-cultured with HepG2 cells in the transwell chambers. LX-2 cells were treated with Na+/taurocholate in different concentrations. Western blot was used to evaluate the expression of alpha smooth muscle actin (α-SMA), type I collagen, and Toll-like receptor 4 (TLR4) in LX-2 cells.

**Results:**

Concentrations of 12 bile acids in serum of patients and healthy volunteers were determined with LC-MS successively. Principal component analysis, fold change analysis and heatmap analysis identified taurocholic acid (TCA) to be the most changed bile acid. Pathway analysis showed that TCA biosynthesis increased significantly. Spearman rank correlation analysis showed that concentration of TCA in serum of cirrhotic patients was positively associated with Child-Pugh classification. TCA increased the expression of α-SMA, type I collagen, and TLR4 in LX-2 cells. Moreover, the above effect was strengthened when LX-2 cells were co-cultured with HepG2 cells.

**Conclusions:**

Increased TCA concentration in serum of liver cirrhotic patients is mainly due to increased bile acid biosynthesis. TCA is an active promoter of the progression of liver cirrhosis. TCA promoting liver cirrhosis is likely through activating hepatic stellate cells via upregulating TLR4 expression. TCA is a potential therapeutic target for the prevention and treatment of liver cirrhosis.

## Background

Liver cirrhosis is the end stage liver disease resulting from continuous intra-hepatic inflammation and extracellular matrix (ECM) accumulation caused by uncontrolled chronic liver diseases. Liver cirrhosis is a global health problem. One epidemiological study conducted in US veterans showed that the prevalence of liver cirrhosis in 2013 was 1.06%, and the prevalence of liver cirrhosis had doubled from 2001 to 2013 [1]. Due to high prevalence of hepatitis B virus (HBV) infections, liver cirrhosis is also a common disease in China [2]. Moreover, liver cirrhosis can cause complications including variceal bleeding, hepatic encephalopathy and hepatorenal syndrome, which are life-threatening to cirrhotic patients. One systemic analysis estimated that global death attributed to liver cirrhosis was over 1 million [3]. Thus liver cirrhosis has rendered a great burden on health care system globally. To date, the optimal prevention and treatment of liver cirrhosis mainly depends on curing or controlling the primary diseases including hepatitis B, hepatitis C, alcoholic liver disease (ALD), primary biliary cholangitis (PBC), primary sclerosing cholangitis (PSC) and autoimmune hepatitis (AIH). Moreover, most chronic liver diseases can only be controlled but not cured. Risk factors affecting the development and progression of liver cirrhosis are multifactorial. Treatment of liver cirrhosis should be comprehensive. So identifying novel risk factors and potential therapeutic targets for prevention and treatment of liver cirrhosis is of great significance to both clinicians and drug developers.

Bile acids are synthesized in hepatocytes by cytochrome P450 (CYP) from cholesterol through classical and alternative pathways [4, 5]. In the classical pathway, cholesterol is hydroxylated by CYP7A1, CYP8B1, and CYP27A1 and converted to cholic acid (CA) and chenodeoxycholic acid (CDCA). In the alternative pathway, cholesterol is hydroxylated by CYP7A1 to produce 27-hydroxycholesterol, 27-hydroxycholesterol is then converted to CDCA through 7α-hydroxylation by CYP7B1. Bile acyl-CoA synthetase (BACS) and bile acid-CoA:amino acid Nacyltransferase (BAAT) subsequently conjugate taurine or glycine to CA or CDCA to produce taurochenodeoxycholic acid (TCDCA), glycocholic acid (GCA), taurocholic acid (TCA) and glycochenodeoxycholic acid (GCDCA). Because CA, CDCA, TCDCA, GCA, TCA and GCDCA are all synthesized in hepatocytes, these bile acids are termed primary bile acids. Primary bile acids are secreted by hepatocytes into intestinal lumen, and metabolized by enzymes of intestinal bacteria to form secondary bile acids.

Because cholestasis is very common in end stage liver cirrhosis, researchers postulated that serum bile acids might be associated with progression of liver cirrhosis. Metabolomic study can reveal difference of the profiling of bile acids between patients and healthy controls. One metabolomic study indicated that TCA, TCDCA, GCA and glycoursodeoxycholic acid (GUDCA) were the most elevated bile acids in serum of liver cirrhotic patients, and concentrations of these bile acids were positively correlated with Child–Pugh scores [6]. Another metabolomic study showed that TCA, TCDCA, GCDCA, GCA, GUDCA and CDCA in the serum of acute decompensated cirrhotic patients were significantly higher than those in the serum of patients with compensated cirrhosis, and bile acids could serve as makers for risk stratification of cirrhotic patients to develop new onset acute decompensation [7]. However, previous metabolomic studies only evaluated diagnostic and prediction value of specific bile acid in progression of liver cirrhosis, and did not explore and verify specific bile acid to be a potential therapeutic target for liver cirrhosis. The present study aims to identify the specific bile acid and explore its possible mechanisms in promoting liver cirrhosis, and to find a potential therapeutic target for liver cirrhosis.

## Methods

### Clinical samples

Blood samples were collected from 32 patients with liver cirrhosis in the First Affiliated Hospital of Dalian Medical University from March 2013 to March 2015. Blood samples were also collected from 27 healthy volunteers in healthy examination center of the First Affiliated Hospital of Dalian Medical University during the same period. Before blood collection, all the patients and healthy volunteers fasted overnight. Venous blood was collected in the morning, then serum was collected through centrifugation of venous blood. All serum samples were stored at − 80 °C. Diagnosis of cirrhosis was based on a combination of clinical manifestations, laboratory tests and imaging presentations (typical cirrhotic morphological changes, splenomegaly and portal hypertension) in CT scanning or MRI scanning. Hepatocellular carcinoma, intrahepatic cholangiocarcinoma, carcinomas outside of liver, heart failure, renal diseases and metabolic diseases were excluded from our study. All the healthy volunteers were free of liver diseases, heart diseases, renal diseases and metabolic diseases as verified by laboratory tests and ultrasonography. Age, gender, clinical data, laboratory data and imaging data were retrieved from medical records.

### Ethics, consent and permissions

The collections of human serum samples were approved by the Ethics Committee of First Affiliated Hospital of Dalian Medical University (No:LCKY2016–34). And written informed consent was obtained from each cirrhotic patient and healthy volunteer.

### Target bile acid detection

Bile acid standards including lithocholic acid (LCA), hyodeoxycholic acid (HDCA), CDCA, deoxycholic acid (DCA), ursodeoxycholic acid (UDCA), CA, GCA, taurolithocholic acid (TLCA), TCDCA, taurodeoxycholic acid (TDCA), tauroursodeoxycholic acid (TUDCA) and TCA were purchased from Sigma. Bile acid standards were diluted to different gradient concentrations. The collected serum samples stored at − 80 °C were thawed at 4 °C. Each 50 μL serum sample mixed with 10 μL internal standard solution and 300 μL cold protein precipitation liquid (a methanol solution containing 0.1% ammonia) was centrifuged, and 200 μL supernatant was collected and dried under nitrogen. Each dried bile acid extract was dissolved with 50 μL methanol, and then was filtered. Liquid chromatography of Waters I-Class coupled to Waters Xevo TQ-S (IVD) mass spectrometer with an ESI source was used to analyze each bile acid extract and the diluted bile acid standards. Chromatographic separation was performed using ACQUITY UPLC BEH Phenyl Column (2.1 × 50 mm, 2.5 μm). The injection volume of sample was 5 μl. Quality control samples were prepared by mixing all of the dissolved bile acid extracts. During LC-MS analysis, one quality control sample was utilized to ensure data quality every 30 injections. Multiple reaction monitor (MRM) was used to collect data. Standard reference curves were depicted with diluted gradient concentrations of bile acid standards and the corresponding peak areas. The quantitative determination of 12 bile acids in human serum samples was calculated from the corresponding standard reference curves.

### Cell culture

Hepatic stellate cell line LX-2 was purchased from the Cell Bank of the Xiangya Central Experiment Laboratory of Central South University (Changsha, China). Human hepatoma cell line HepG2 was purchased from American Type Culture Collection (ATCC, Manassas, VA). Cells were cultured in Dulbecco’s Modified Eagle’s Medium (DMEM) (Gibco) supplemented with 10% fetal bovine serum (FBS) (Gibco) and antibiotics (100 IU/ml penicillin and 100 mg/ml streptomycin) in an incubator with humidified air containing 5% CO2 at 37 °C. Co-culture of the LX-2 and HepG2 cells in the transwell chambers was according to the method in a previous study [8].

### Cell proliferation assay

LX-2 cells were plated into 96-well plates at a density of 2 × 10^4^ cells/ml per well and incubated for 24 h, then incubated with Na+/taurocholate (Sigma) in different concentrations (50 μM, 75 μM, 100 μM and 150 μM) for another 24 h. Phosphate buffered saline (PBS) was as the control solution in cell proliferation assay. Cell proliferation was analyzed with Cell Counting Kit (Dojindo) according to the manufacturer’s protocols.

### Western blotting assay

LX-2 cells were treated with Na+/taurocholate (Sigma) (50 μM and100μM) dissolved in DMEM without FBS for 24 h. LX-2 cells co-cultured with HepG2 cells were also treated with Na+/taurocholate (Sigma) (50 μM and 100 μM) dissolved in DMEM without FBS for 24 h. Total cellular proteins were extracted with a protein exaction kit (Beyotime Biotechnology) according to the manufacturer’s protocols. BCA Protein Assay Kit (Beyotime Biotechnology) was used for quantification of exacted proteins. After separated with SDS-PAGE, the proteins were transferred to a PVDF membrane. The PVDF membrane was blocked with 5% skim milk in Tris-buffered saline containing 0.05% Tween-20 (TTBS). The membranes were then incubated with the primary antibodies against Toll-like receptor4 (TLR4) (Santa Cruz Biotechnology), alpha Tubulin (α-Tubulin) (Proteintech), Collagen Type I (Proteintech) and smooth muscle actin (α-SMA) (Proteintech) overnight at 4 °C, and subsequently incubated with the secondary antibody for 2 h at 37 °C. Protein band was detected with the enhanced chemiluminescence (Advansta) method and imaged with a Bio-Rad ChemiDoc MP imaging system. Intensity of the α-Tubulin band was as the internal reference.

### Statistical analyses

Principal component analysis (PCA), fold change analysis, partial least squares discriminant analysis (PLS-DA), heatmap analysis and pathway analysis were used to analyze bile acid metobolomic data of human serum samples. PCA, fold change analysis, PLS-DA, heatmap analysis and pathway analysis were conducted with MetaboAnalyst (http://www.metaboanalyst.ca/) according to the manual of analysis provided in the website [9]. Unpaired t test was employed to determine the difference of ages between cirrhotic patients and healthy controls. Chi-square test was utilized to determine the difference of gender between cirrhotic patients and healthy controls. Spearman correlation analysis was used to evaluate the association between concentrations of bile acids and Child-Pugh classification. Cell experimental data were presented as mean and standard deviation (SD). One-way ANOVA analysis and least significant difference (LSD) test were employed to determine the difference of means among three groups. Unpaired t test was employed to determine the difference of means between two groups. *P* < 0.05 was considered statistically significant. One-way ANOVA analysis, Chi-square test, Spearman correlation and unpaired t test were performed with the SPSS16.0 statistical software package (SPSS Inc., Chicago, IL, USA).

## Results

### Characteristics of liver cirrhotic patients and healthy controls

Thirty two liver cirrhotic patients and 27 healthy controls were enrolled in this study. Age and gender distributions between cirrhotic patients and healthy controls were not statistically significant. The causes of liver cirrhosis included HBV infection (13 patients), ALD (six patients), PBC (seven patients). And six patients were diagnosed with cryptogenic cirrhosis. Twelve cirrhotic patients were classified as Child-Pugh A, 17 cirrhotic patients were classified as Child-Pugh B, and three cirrhotic patients were classified as Child-Pugh C. The detailed characteristics of liver cirrhotic patients and healthy controls were presented in Table 1.

**Table 1:** Characteristics of liver cirrhotic patients and healthy controls

	Liver cirrhosis	Healthy control	*P*
Male/Female	18/14	9/18	0.134
Age(years)	59.00 ± 12.92	51.78 ± 18.99	0.089
Child Pugh class A	12	–	–
Child Pugh class B	17	–	–
Child Pugh class C	3		–
Hepatitis B virus infection	13	–	–
Alcoholic liver disease	6	–	–
Primary biliary cholangitis	7	–	–
Cryptogenic cirrhosis	6	–	–

### Target bile acid metabolomic analysis

**Fig. 1:**
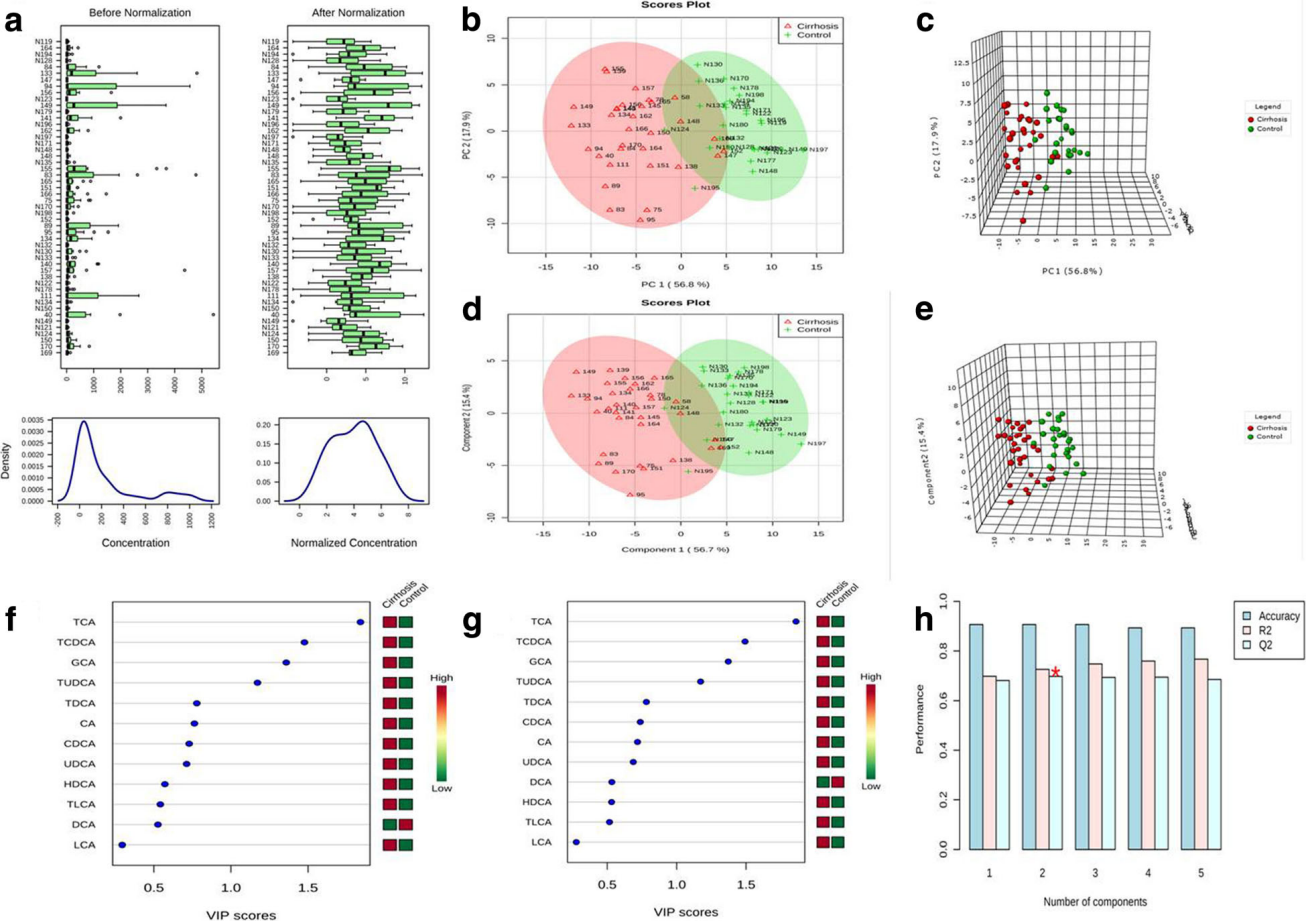
Normalization of original data, PCA and PLS-DA, importance in projection (VIP) analysis and cross validation of the optimal number of components of classification. **a** The appearance of characteristic graphical summary of data became bell-shaped distribution after a log transformation. **b** and **c** Both two dimension scores plot and three dimension scores plot of PCA indicated that there was a distinguished classification between the observation clustering of liver cirrhosis group and that of healthy control group. **d** and **e** Both two dimension scores plot and three dimension scores plot of PLS-DA indicated that there was a distinguished classification between the observation clustering of liver cirrhosis group and that of healthy control group. **f** and **g** VIP analysis of PLS-DA indicated that TCA was the most important metabolite in component one and component two. **h** Cross validation analysis indicated that two components model was the optimal model

Qualification and quantification of 12 bile acids in serum of liver cirrhotic patients and healthy controls were performed with LC-MS successively. Data normalization was recommended by MetaboAnalyst to reduce any systematic bias within a given data set and to improve overall data consistency so that meaningful biological comparisons can be made, and bell-shaped distribution of the appearance of characteristic graphical summary indicated the proper normalization [9]. In our study, the appearance of characteristic graphical summary of data became bell-shaped distribution after a log transformation (Fig. 1a).

PCA was used to visualize general clustering and trend of bile acids between groups. Both two dimension scores plot and three dimension scores plot showed that there was a distinguished classification between the observation clustering of liver cirrhosis group and that of healthy control group (Fig. 1b and c). PLS-DA was able to identify the most important biomarker between groups. PLS-DA also discriminated the observation clustering of liver cirrhosis group from that of healthy control group (Fig. 1d and e). PLS-DA showed that two components model was the optimal model (Fig. 1h). Importance in projection (VIP) analysis of PLS-DA indicated that TCA was the most important metabolite in component one and component two (Fig. 1f and g).

**Fig. 2:**
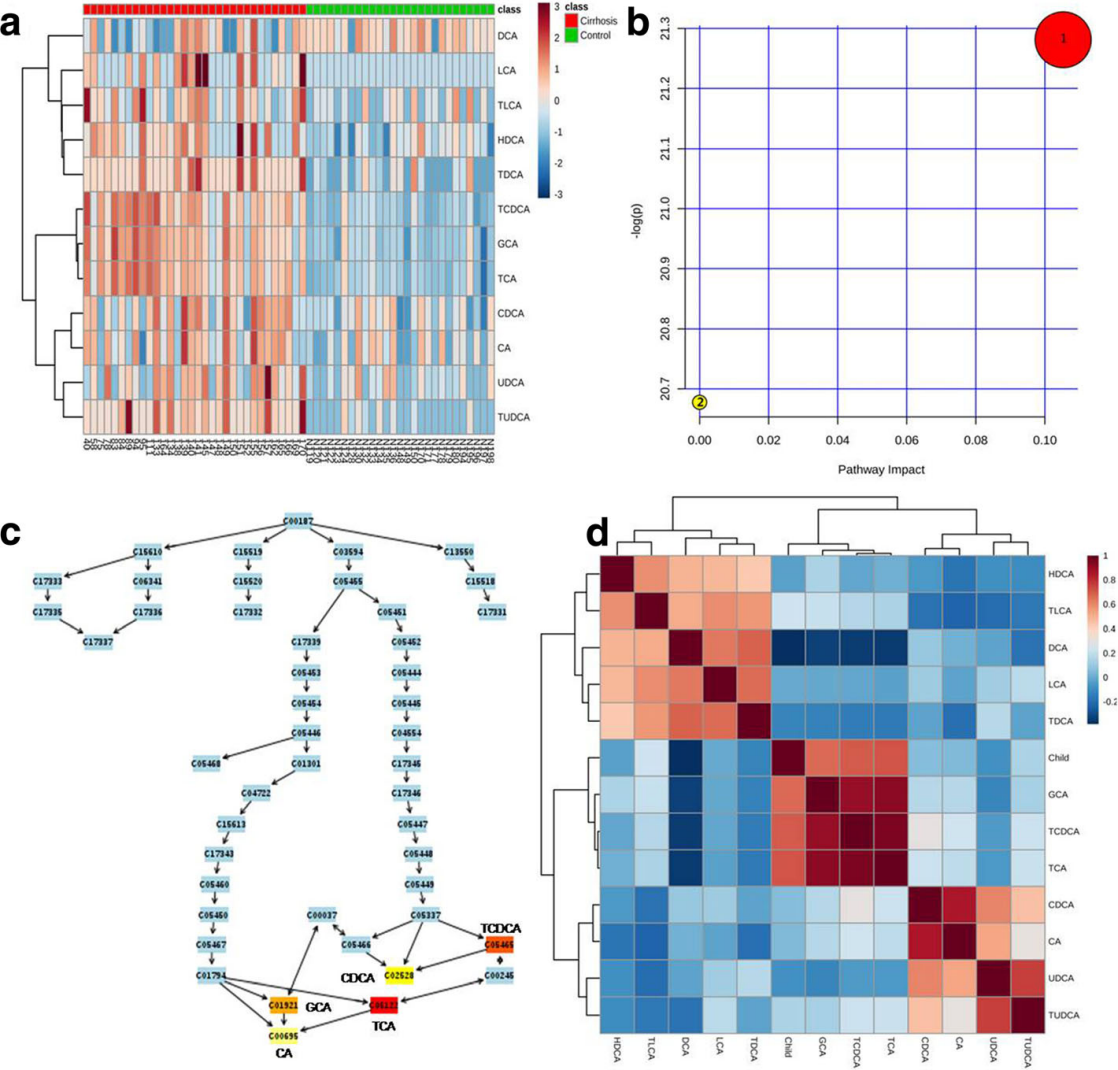
Heatmap analysis, summary of pathway effect, compound impact on pathway and Spearman correlation. **a** Heatmap analysis indicated that TCA, TCDCA, TUDCA, GCA, UDCA, CDCA, CA, TLCA, TDCA, HDCA and LCA were increased in liver cirrhosis as compared with healthy controls. **b** Pathway analysis showed that primary bile acid biosynthesis was increased in liver cirrhosis. 1: bile acid biosynthesis. 2: taurine and hypotaurine metabolism. **c** Compound impact analysis implied that TCA impacted most in the increased primary bile acid biosynthesis in liver cirrhosis. **d** Spearman correlation analysis indicated that concentrations of TCA, GCA and TCDCA were significantly positively correlated with Child-Pugh classification (*P* < 0.0001). And concentrations of LCA, HDCA, CDCA, UDCA, CA, TLCA, TDCA and TUDCA were not correlated with Child-Pugh classification (*P* > 0.05)

Heatmap analysis showed that TCA, TCDCA, TUDCA, GCA, UDCA, CDCA, CA, TLCA, TDCA, HDCA and LCA were increased in liver cirrhotic patients as compared with healthy controls (Fig. 2a). Fold change analysis indicated that TCA was the most changed bile acid in liver cirrhotic patients, and DCA was the least changed bile acid in liver cirrhotic patients, as illustrated in Table 2. Unpaired t test showed that all the 12 bile acids in the liver cirrhosis group were significantly changed as compared with those in the control group, as illustrated in Table 3.

**Table 2:** Fold change of bile acid in liver cirrhosis

	TCA	TCDCA	TUDCA	GCA	UDCA	CDCA	CA	TLCA	TDCA	HDCA	LCA	DCA
Fold Change(FC)	76.343	47.358	32.897	27.335	12.131	6.1778	5.714	4.9992	4.7731	3.4723	2.3062	1.1365
log2(FC)	6.2544	5.5655	5.0399	4.7727	3.6006	2.6271	2.5145	2.3217	2.2549	1.7959	1.2055	0.18464

**Table 3:** Comparison of concentration of twelve bile acids between liver cirrhosis and healthy control

	t	*P*	-log10(*P*)	FDR
TCA	10.11	2.51 × 10^−14^	13.6	2.16 × 10^−13^
TCDCA	10.013	3.59 × 10^−14^	13.445	2.16 × 10^−13^
GCA	9.7355	9.95 × 10^−14^	13.002	3.98 × 10^−13^
TUDCA	8.6658	5.45 × 10^−12^	11.264	1.63 × 10^−11^
TDCA	7.2181	1.38 × 10^−9^	8.8608	3.31 × 10^−09^
HDCA	4.69	1.75 × 10^−5^	4.757	3.50 × 10^−05^
LCA	4.2705	7.47 × 10^−5^	4.1266	0.00012808
UDCA	3.4311	0.001125	2.9489	0.0016874
CDCA	3.2898	0.0017223	2.7639	0.0022964
TLCA	3.2346	0.0020289	2.6927	0.0024346
CA	3.0365	0.0036053	2.4431	0.003933
DCA	−2.3474	0.022403	1.6497	0.022403

FDR value is the false discovery rate adjusted *P* value

Pathway analysis was also used to identify the significantly changed metabolic pathway in bile acid metabolization according to the KEGG pathway database. Impact value more than 0.1 and hits value more 3 were used as the threshold to identify the significantly changed metabolic pathway [10]. Pathway analysis showed that primary bile acid synthesis was increased in liver cirrhosis (Fig. 2b and Table 4). Moreover, TCA was the most important metabolite in the increased primary bile acid synthesis in liver cirrhosis (Fig. 2c).

**Table 4:** Pathway analysis of twelve bile acids in liver cirrhosis compared with healthy volunteers

Pathway name	Hit	*P*	-log(*P*)	FDR	Impact
Primary bile acid biosynthesis	5	5.72 × 10–10	21.281	1.05 × 10–9	0.10527
Taurine and hypotaurine metabolism	1	1.05 × 10–9	20.677	1.05 × 10–9	0

Hit means the matched number of bile acid in metabolization pathway; The *P* value is calculated from the enrichment analysis; Impact value is calculate from pathway topography analysis; FDR value is the false discovery rate adjusted *P* value

### Spearman correlation analysis between child-Pugh classification and concentrations of twelve bile acids

Spearman correlation analysis showed that concentrations of TCA, GCA and TCDCA were significantly positively correlated with Child-Pugh classification (*P* < 0.0001) (Fig. 2d). Spearman correlation analysis indicated that concentrations of LCA, HDCA, CDCA, UDCA, CA, TLCA, TDCA and TUDCA were not correlated with Child-Pugh classification (*P* > 0.05) (Fig. 2d).

### Effects of TCA on proliferation of hepatic stellate cell

**Fig. 3:**
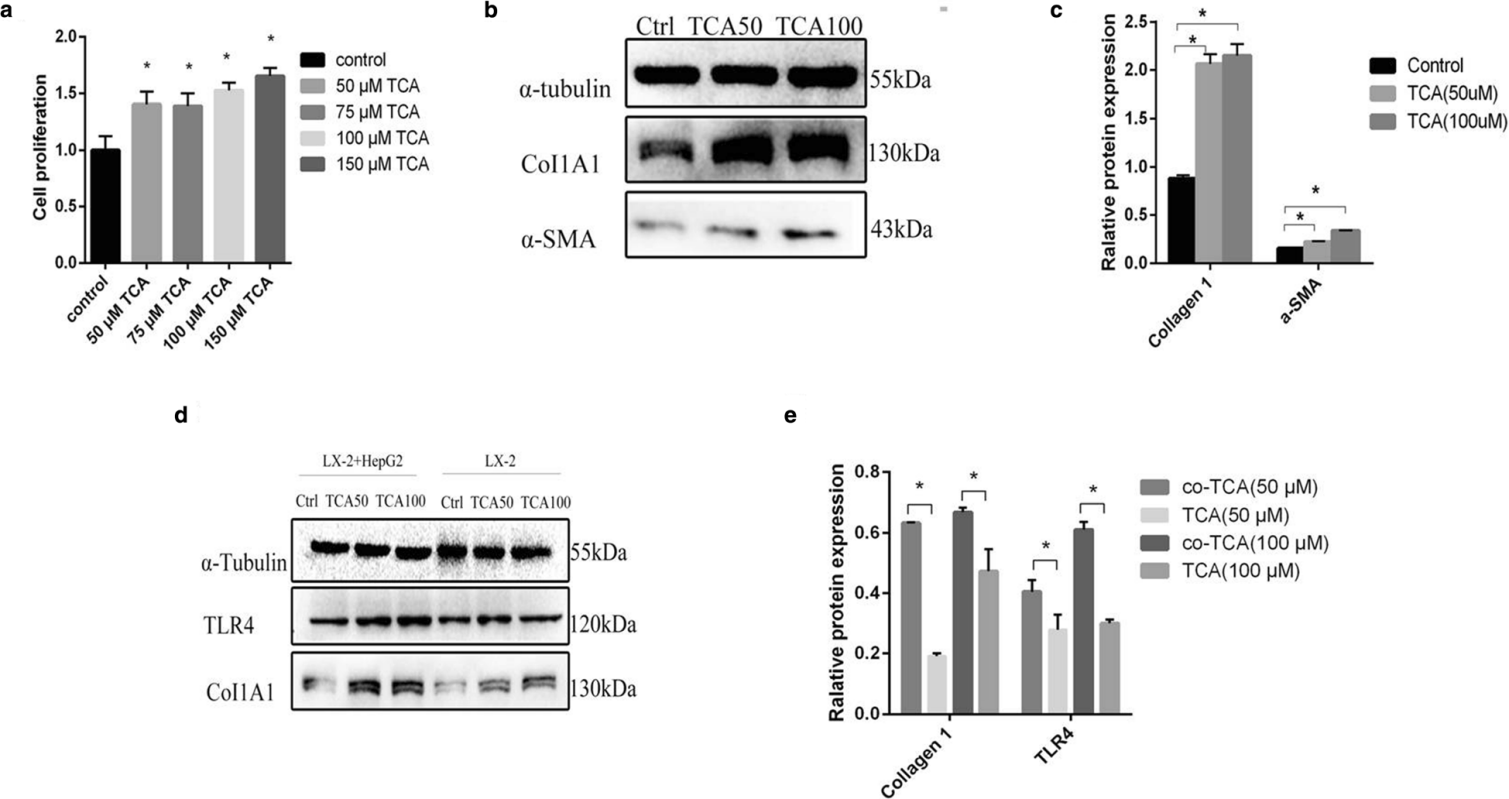
Cell experiment. **a** Cell proliferation assay indicated that growth rate of LX-2 cells treated with the different concentrations of Na+/taurocholate were increased compared to that of the control group. Moreover, the effect was dose-dependent. *P < 0.05 compared with control. **b** and **c** Western blot indicated that expression of Collagen Type I and α-SMA was increased by TCA treatment as compared with the control. Moreover, effect of TCA on the expression of Collagen Type I and α-SMA was also dose-dependent. TCA50 means 50 μM Na+/taurocholate, and TCA100 means 100 μM Na+/taurocholate. *P < 0.05 compared with control. **d** and **e** Western blot indicated that the effect of TCA on Collagen Type I and TLR4 expressions in co-culture groups was more significant than that in mono-culture group. TCA50 means 50 μM Na+/taurocholate, and TCA100 means 100 μM Na+/taurocholate. *P < 0.05 compared with mono-culture group

In order to evaluate the effect of TCA on liver cirrhosis, we evaluated the effect of TCA on proliferation of LX-2 cells. Proliferation assay showed that TCA (50 μM, 75 μM, 100 μM and 150 μM) increased proliferation of LX-2 cells significantly (Fig. 3a). Moreover, the effect of TCA on proliferation of LX-2 was dose-dependent (Fig. 3a).

### Effects of TCA on expression of collagen type I, α-SMA and TLR4

Collagen Type I expression and α-SMA expression were indicators of the activation level of stellate cells [11]. According to the concentrations of TCA in proliferation assay of LX-2 cells, we selected 50 μM and 100 μM TCA to treat LX-2 cells. Western blot showed that expression of Collagen Type I and α-SMA was increased by TCA treatment as compared with the control (Fig. 3b and Fig. 3c). Moreover, effect of TCA on the expression of Collagen Type I and α-SMA was also dose-dependent (Fig. 3b and c).

Accumulating evidences showed that TLR4 promoted hepatic stellate cell activation by down-regulating the TGF-β pseudoreceptor BAMBI in order to render hepatic stellate cell sensitive to TGF-β signaling [12, 13]. So we evaluated the effect of TCA on expression of TLR4 of LX-2 cells. Western blot showed that expression of TLR4 was increased by TCA treatment as compared with the control, and the effect was dose-dependent (Fig. 3d and e).

In order to mimic the interaction between hepatocytes and hepatic stellate cells in liver. We co-cultured LX-2 cells with HepG2 cells. Western blot showed that the effect of TCA on Collagen Type I and TLR4 expressions in co-culture group was more significant than that in mono-culture group (Fig. 3d and e).

## Discussion

Cholestasis especially intra-hepatic cholestasis is very common in various liver diseases [14]. The mechanism of cholestasis including inflammatory damaging of biliary canaliculi and downregulation of critical bile acid transporters of hepatocytes [14–16]. As liver cirrhosis is the end stage of chronic liver diseases, cholestasis is also a prominent manifestation of liver cirrhosis. Moreover, regenerative nodules surrounded by fibrous septa in liver cirrhosis can compress intrahepatic biliary trees to further aggravate cholestasis. Bile acid is an important component of bile and cholestasis can cause serum bile acid elevation [14]. So researchers postulate that bile acid might be associated with progression of liver cirrhosis, and have diagnostic value in classification of stages of liver cirrhosis.

A study conducted in 1986 showed that total serum-conjugated primary bile acids were more sensitive than conventional liver function test in evaluating prognosis of liver cirrhosis [17]. However, this study did not evaluate specific bile acid in diagnosis and prognosis of liver cirrhosis because of technology limitations. Recently, bile acid profiling method was used to study bile acid. One urinary metabolomic study showed that glycocholate 3-glucuronide, taurohyocholate, TCA, glycolithocholate 3-sulfate, and GUDCA were markedly elevated in hepatitis B-induced liver cirrhosis compared with healthy controls [18]. Another metabolomic study revealed that TCA, TCDCA, GCA and GUDCA were the most elevated bile acids in serum of liver cirrhotic patients, and concentrations of these bile acids were positively correlated with Child–Pugh scores [6]. A recent metabolomic study further showed that TCA, TCDCA, GCDCA, GCA, GUDCA and CDCA in the serum of acute decompensated cirrhosis patients were significantly higher than those in the serum of patients with compensated cirrhosis, and bile acids could serve as maker for risk stratification of cirrhotic patients to develop new onset acute decompensation [7]. Our study indicated that TCA, TCDCA, TUDCA and GCA were the four most changed bile acids in liver cirrhosis, and TCA, TCDCA and GCA were positively correlated with Child-Pugh classification. Correspondingly, CA and DCA were the least changed bile acids, and were not correlated with Child-Pugh classification. The results of our study confirm the findings of previous studies. Moreover, when we observe results of the previous study and the present study, we find that TCA is the most changed bile acid in liver cirrhosis. Furthermore, previous studies did not conduct pathway analysis of bile acids. So we conducted a metabolomic pathway analysis of bile acid in our study, the results showed that primary bile acid biosynthesis was increased and TCA was the most important metabolite in the increased primary bile acid synthesis in liver cirrhosis. As one study revealed that fecal bile acids were decreased in liver cirrhosis, the attenuated feedback of bile acid enterohepatic circulation on primary bile acid biosynthesis might account for this phenomenon [19].

Although TCA is elevated in serum of liver cirrhotic patients, whether elevated TCA in liver cirrhosis is a promoting factor in progression of liver cirrhosis needs to be elucidated. So we conducted cell experiments to study the effect of TCA on hepatic stellate cell. Cell experiment showed that TCA increased proliferation of LX-2 cells and upregulated the expression of α-SMA and type I collagen of LX-2 cells, which implied that TCA was able to activate hepatic stellate cell to promote progression of liver cirrhosis. Moreover, the effect of TCA on hepatic stellate cell is dose-dependent, which accounts for the findings in the human metabolomic study that concentration of TCA is positively correlated with stages of liver cirrhosis. Moreover, the above effect of TCA on LX-2 cells was strengthened when LX-2 cells was co-cultured with HepG2 cells. These results support TCA as a promoting factor in liver cirrhosis, not just a manifestation of liver cirrhosis.

TLR4 signaling pathway plays an important role in the development of liver cirrhosis. Molecular epidemiology studies have revealed that polymorphisms of TLR4 gene (T399I and D299G) can affect the susceptibility of HCV infected patients to develop liver cirrhosis [20, 21]. Cell study also proves that hepatic stellate cell transfected with T399I and D299G mutations displays decreased cytokine and chemokine release and BAMBI downregulation in response to lipopolysaccharides (LPS) [22]. TLR4 promotes hepatic stellate cell activation by down-regulating the TGF-β pseudoreceptor BAMBI in order to render hepatic stellate cell sensitive to TGF-β signaling [12, 13]. Additionally, TLR4 signaling pathway orchestrates with increased intestinal permeability in liver cirrhosis. Various case control studies indicates that intestinal permeability is increased in liver cirrhosis compared with healthy controls [23–27]. With dysfunction of intestinal barrier, intestinal bacteria and LPS entering liver via portal vein system would be increased. LPS then activates hepatic stellate cell via TLR4. Activated hepatic stellate cell produces ECM in liver to remodel hepatic lobuli [28]. Moreover, activated hepatic stellate cells are recruited around sinusoidal vessels to increase intrahepatic vascular resistance to blood flow [29–31]. Activated hepatic stellate cell displaying decreased response to vasodilators also increases vascular resistance [32]. Therefore, TLR4 signaling in hepatic stellate cell plays important roles in both liver remodeling and portal hypertension development.

We evaluated the effect of TCA on expression of TLR4 of LX-2 cells. We found that TCA increased the expression of TLR4 of LX-2 cells and the effect was dose-dependent. Furthermore, in order to mimic the environment of hepatocyte and hepatic stellate cell interaction, LX-2 cells were co-cultured with HepG2 cells and the results showed that co-culture increased the effect of TCA on the expressions of TLR4 of LX-2 cells. Thus, TCA activates hepatic stellate cell via upregulating TLR4 signaling.

However, when evaluating the findings of our study we should be cautious. Our study has some limitations. First, we did not estimate the sample size in our metabolomic study, so the possibility of lack of power to come to a definite conclusion could not be ruled out. So future studies with large sample size are needed to validate the findings of our study. Second, our in vitro study only evaluated the effect of TCA on expression of TLR4 of LX-2 cells. Future studies can evaluate the detailed mechanism of TCA activating hepatic stellate cell via TLR4 signaling with transgenic animal studies.

## Conclusion

The present study provides evidence of TCA as an active promoter in liver cirrhosis. Increased TCA concentration in cirrhosis is mainly due to increased bile acid biosynthesis. TCA is an active promoter of the progression of liver cirrhosis not just a bystander. The mechanisms of TCA promoting liver cirrhosis are likely through activating hepatic stellate cell via TLR4 pathways. TCA is a potential therapeutic target for the prevention and treatment of liver cirrhosis.

## Abbreviations

AIH: Autoimmune hepatitis
ALD: Alcoholic liver disease
BAAT: Bile acid-CoA
BACS: Bile acyl-CoA synthetase
CA: Cholic acid
CDCA: Chenodeoxycholic acid
CYP: Cytochrome P450
DCA: Deoxycholic acid
DMEM: Dulbecco’s Modified Eagle’s Medium
ECM: Extracellular matrix
FBS: Fetal bovine serum
GCA: Glycocholic acid
GCDCA: Glycochenodeoxycholic acid
HBV: Hepatitis B virus
HDCA: Hyodeoxycholic acid
HSC: Hepatic stellate cell
LCA: Lithocholic acid
LSD: Least significant difference
MRM: Multiple reaction monitor
PBC: Primary biliary cholangitis
PBS: Phosphate buffered saline
PCA: Principal component analysis
PLS-DA: Partial least squares discriminant analysis
PSC: Primary sclerosing cholangitis
SD: Standard deviation
TCA: Taurocholic acid
TCDCA: Taurochenodeoxycholic acid
TDCA: Taurodeoxycholic acid
TLCA: Taurolithocholic acid
TLR4: Toll-like receptor4
TTBS: Tris-buffered saline containing 0.05% Tween-20
TUDCA: Tauroursodeoxycholic acid
UDCA: Ursodeoxycholic acid
VIP: Importance in projection
α-SMA: Alpha smooth muscle actin
